# Acquired right-sided diaphragmatic hernia in a patient with retroperitoneal hydatidosis: a case report and review of the literature

**DOI:** 10.1186/s13256-021-02836-z

**Published:** 2021-06-18

**Authors:** Dhuha Boumarah, Ali Alsinan, Omar Alothman, Omran AlDandan, Saeed Alshomimi

**Affiliations:** 1 Surgery Department, King Fahd Hospital of the University, Imam Abdulrahman Bin Faisal University, Khobar, Saudi Arabia; 2 Radiology Department, King Fahd Hospital of the University, Imam Abdulrahaman Bin Faisal University, Khobar, Saudi Arabia

**Keywords:** Diaphragmatic hernia, Hydatid disease, Retroperitoneal cyst, Laparoscopic surgery, Case report

## Abstract

**Background:**

Diaphragmatic hernia is primarily congenital in origin and has potentially devastating pulmonary complications. Acquired diaphragmatic hernia as a complication of hydatid disease remains a rare clinical entity. Retroperitoneal hydatidosis, in particular is an exceptionally rare cause behind a similar presentation. This paper aims to present the first case of acquired diaphragmatic hernia likely caused by eroding retroperitoneal hydatid cysts and provide a succinct literature review regarding the causative association between hydatid disease and diaphragmatic defects.

**Case presentation:**

A 71-year-old Saudi man, with a history of hydatid disease involving several areas including the retroperitoneum, presented with multiple episodes of shortness of breath and abdominal pain of 10 months’ duration. Computed tomography scans of the chest and abdomen demonstrated the presence of a large diaphragmatic defect, with herniation of bowel loops into the chest cavity. Initially, the patient underwent a diagnostic laparoscopy which was then converted to a posterolateral thoracotomy to repair the defect.

**Conclusions:**

The ability of hydatid disease to involve several body organs makes diagnosis and management of resultant complications a challenge in some cases, like ours. Knowledge about a reported rare complication could enable early detection and management to avoid serious complications, including abdominal viscera incarceration and strangulation.

## Background

Diaphragmatic hernia (DH) is a transdiaphragmatic protrusion of abdominal viscera into the thoracic cavity through a diaphragmatic defect. It is primarily congenital in origin and has potentially devastating pulmonary complications. Anatomically, it is classified into three types: Bochdalek, Morgagni and esophageal hiatal hernias. Although Bochdalek hernia is the most common type among neonates, adult onset is extremely rare, with less than 100 cases reported in the literature [1]. Nevertheless, it is a potentially life-threatening condition, as it can lead to abdominal viscera incarceration and even strangulation, which account for an overall mortality rate reaching 31% [2]. Although cystic hydatid disease can virtually infect any body organ and has a great tendency to infect the liver, demonstrated in 70% of cases, acquired diaphragmatic hernia (ADH) as a complication of hydatid cysts remains a rare entity [3]. Herein, we report a case of nontraumatic, right-sided, DH in an adult patient probably caused by eroding retroperitoneal-hydatid cysts. The case has been reported in accordance with the CARE guidelines.

## Case presentation

A 71-year-old Saudi male patient with a 10-year history of hydatid disease involving the right kidney, retroperitoneum and right inguinal subcutaneous tissue, treated years earlier by a course of albendazole 400 mg daily for 2 months followed by partial nephrectomy, presented with multiple episodes of sudden attacks of shortness of breath and dull abdominal pain for the past 10 months. The attacks were severe enough to require multiple emergency room visits and admissions. The patient did not recall any significant history of trauma or lung infection. There was no history of chronic cough or chronic constipation. He had a significant past medical history of chronic kidney disease, diabetes mellitus, hypertension, renal stones and ischemic heart disease, for which he had undergone percutaneous coronary intervention 2 years earlier, and was started on antiplatelet therapy. The patient had also experienced several thrombotic events affecting his lower limbs in the form of “trash feet,” which were managed at that time without complications.

**Fig. 1:**
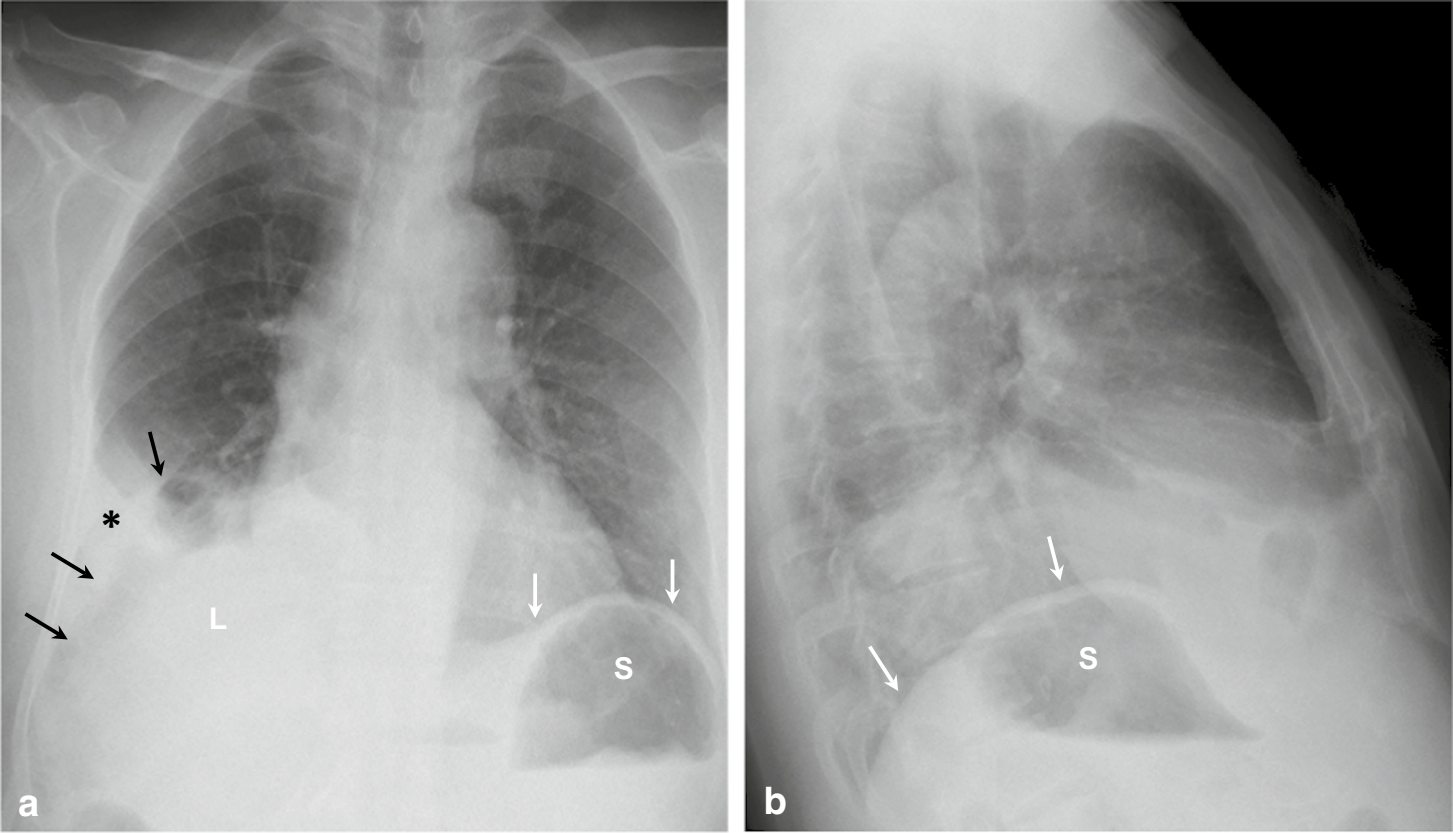
Plain radiograph of the chest: frontal (**a**) and lateral view (**b**) demonstrate non-visualization of left dome of the diaphragm on both views. Note the normal left hemidiaphragm (white arrows in **a** and **b**) with air bubbles within the stomach (S) below. A bowel loop (black arrows in **a**) is seen extending above the liver shadow (L in **a**) at the lower chest. A small pleural effusion (*) is also present

**Fig. 2:**
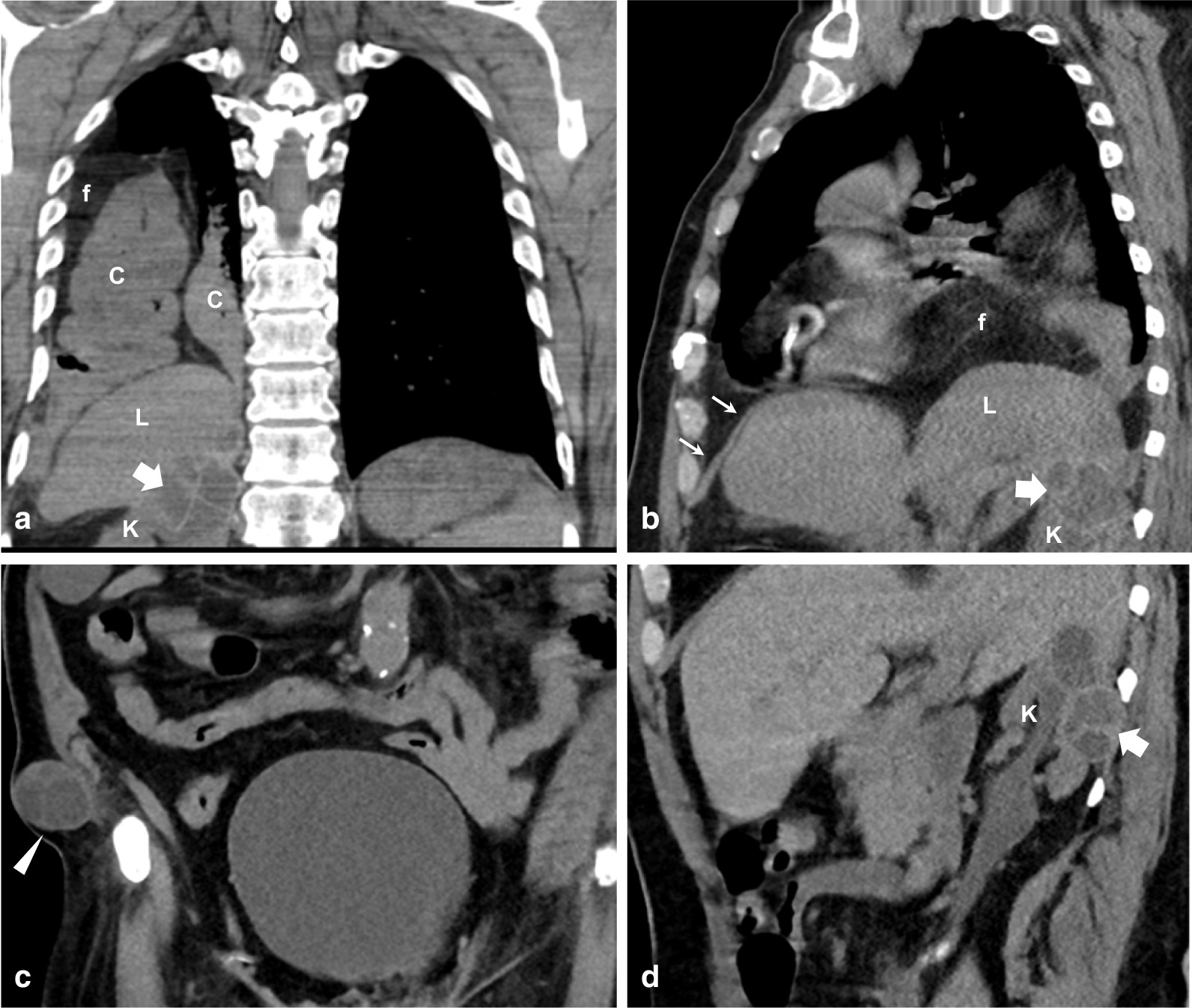
Unenhanced computed tomography scan of the chest (**a** coronal and **b** sagittal) and of the abdomen (**c** coronal and **d** sagittal), done on different dates, showing colonic loops (C in **a**) and accompanying fat (F in **a**, **b**) within the right hemithorax. The anterior portion of the diaphragm is present (thin arrows in **b**) over the liver (L), while the posterior portion is absent. Imaged portions of the upper abdomen show an exophytic multilocular/complex cystic lesion (thick arrows in **a**, **b**, and **d**), arising from the upper pole of the kidney (K) and abutting the liver (L). A similar lesion is seen in the subcutaneous tissue of the lower abdomen (arrowhead in **c**)

Abdominal examination was unremarkable except for a painless, soft swelling over the right inguinal region. Additionally, his routine blood tests and hydatid titer were within normal limits. Chest X-ray (Fig. 1) showed absence of a right diaphragmatic shadow, with bowel loops projecting over the lower chest above the level of the liver. A small pleural effusion was also present. For further evaluation of the condition, computed tomography (CT) scans of the chest and abdomen were performed and clearly demonstrated the presence of multiloculated cystic lesion at the upper pole of the right kidney, as shown in Fig. 2. The lesion was abutting the crus and the dome of the right hemidiaphragm. Further, a large diaphragmatic defect was seen at the posteromedial aspect with herniation of bowel loops and accompanying fat into the chest cavity. Subsequently, collapse of the lower lobe of the lung was evident.

**Fig. 3:**
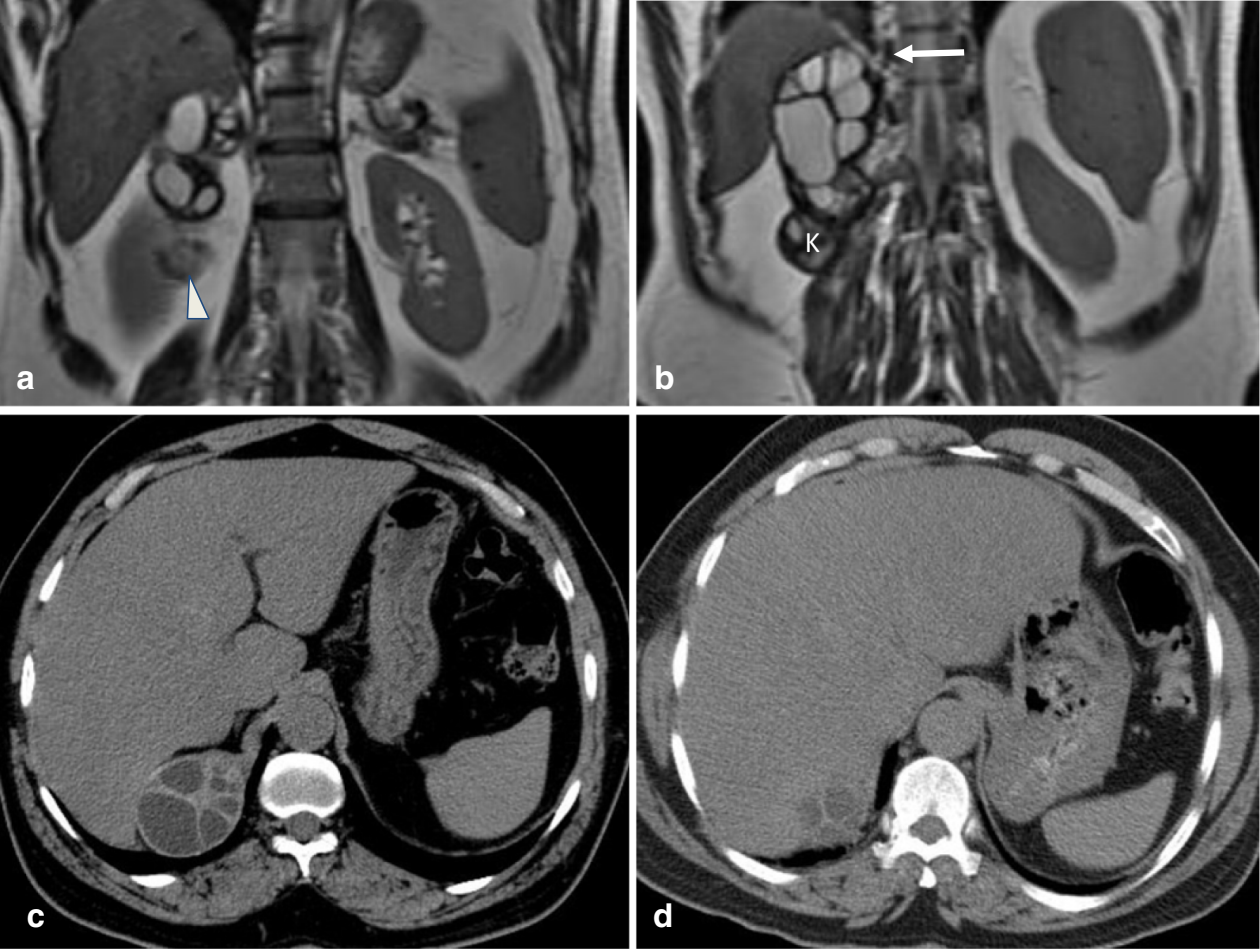
T2-weighted magnetic resonant coronal images (**a**, **b**) and computed tomography axial images (**c**, **d**) show a multiloculated cystic lesion in the retroperitoneum. This abuts the lower surface of the liver and the right crus of the diaphragm (thick white arrow) as well as the posteromedial aspect of the dome. It also abuts the right kidney which is partially visualized (k). A smaller lesion within the kidney is also seen (arrowhead). Note that the dome of the diaphragm is intact on this scan

Ten years earlier, magnetic resonance imaging and CT of the abdomen were performed in an outside hospital and demonstrated that the diaphragm was intact. There was also a larger retroperitoneal multiloculated lesion that abuts the crus of the right hemidiaphragm as well as its dome, notably at the posteromedial aspect. A smaller similar lesion was seen within the right kidney, as illustrated in Fig. 3.

Based on his clinical situation, the decision was made to repair his DH, hopefully through a laparoscopic approach.

**Fig. 4:**
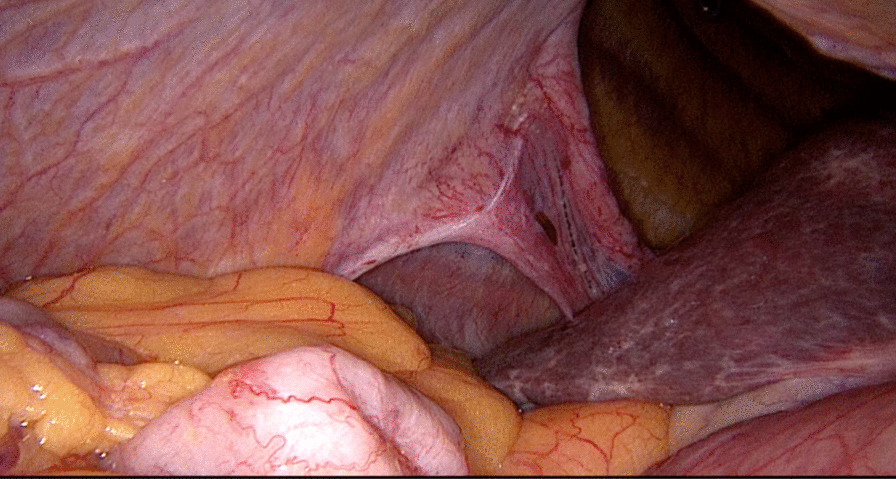
A laparoscopic view showing partial herniation of the abdominal viscera through the two diaphragmatic defects, with absence of hernia sac in both

**Fig. 5:**
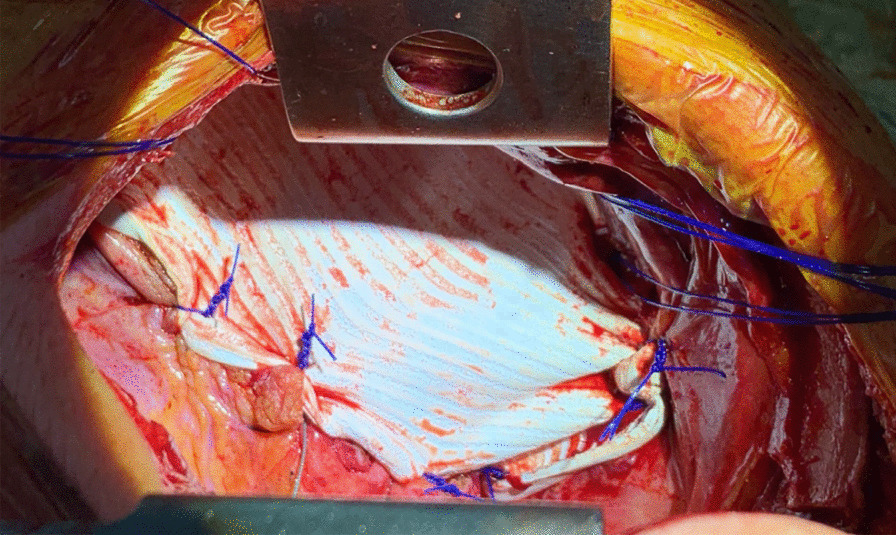
Intraoperative image of the large diaphragmatic defect after repair with Gore-Tex DualMesh

The operation started as a diagnostic laparoscopy where major portions of the small bowel, omental fat, and ascending and transverse colon were found to be partially herniating through two diaphragmatic defects, located posteromedially. The defects were separated from each other by a delicate septum with a total defect size of about 10 × 15 cm, and neither had a hernia sac, as shown in Fig. 4. The diagnosis of right-sided adult Bochdalek hernia was established, and hernial components were successfully reduced back into the abdominal cavity. However, the absence of a posterior anchoring point of the diaphragm made approximation of diaphragmatic edges or mesh implantation difficult to accomplish laparoscopically. Therefore, the approach was changed to posterolateral thoracotomy, where the two orifices were connected to each other, creating a 10 cm defect. The hernia was repaired with Gore-Tex DualMesh (15.0 cm × 19.0 cm × 2.0 mm oval) using a bridging technique fixed with Prolene 0 non-absorbable sutures, as demonstrated in Fig. 5. Postoperatively, the patient was transferred to the intensive care unit and kept intubated. Two days postoperatively, he developed deterioration in liver function, demonstrated in the form of elevated aspartate and alanine aminotransferases reaching 2400 and 2000 IU/L, respectively. CT scan was done and showed perfusion changes; however, hepatic and portal veins and hepatic arteries were all patent, with no evidence of biliary dilatation. Liver enzymes returned to baseline after a few days. The patient was discharged from the hospital in good condition on postoperative day 20.

Currently, 2 years postoperatively, the patient is symptom-free, with significant improvement in terms of his original complaints.

## Discussion

Hydatid disease (HD), or echinococcosis, is a zoonotic disease caused mainly by the parasitic organism *Echinococcus granulosus*. The disease is endemic in several countries of the Middle East, including Saudi Arabia, and is responsible for 1–3 million disability-adjusted life-years annually [4].

The fluid-filled hydatid cysts have the ability to infect several body organs; however, the liver and lungs are the most common sites of involvement, with frequency of 70% and 20%, respectively [3]. The retroperitoneum, in particular, remains an uncommon site for hydatid disease infestation [5]. Cyst rupture and erosions to neighboring structures are potential complications of HD if left untreated. In our case, the multiseptate retroperitoneal lesion was located proximal to the diaphragmatic muscle; consequently, erosions by hydatid cysts probably resulted in ADH.

Interestingly, 34 cases were found in the English literature reporting this causative potential of hydatid disease, none of which was retroperitoneal in position or a consequence of spontaneous erosion, highlighting the uniqueness of the present case [6–12]. Table 1 compares the cases in terms of patient presentation, surgical management and postoperative course.

**Table 1.** A brief summary of hydatid disease cases resulting in a diaphragmatic defect

Author(s)	No. of patients	Year of publication	Demographics (age and sex)	Presenting symptoms	Primary organ involved	Surgical approach	Method of repair	Postoperative course
Bulut *et al*. [6]	1	2017	56 years Male	Dyspnea, RUQ pain and fever	Liver	Thoracotomy	Primary repair	Bronchopleural fistula
Hachim *et al*. [7]	1	2017	34 years Male	RUQ pain, weight loss and fatigue	Liver	Laparotomy	A unique approach was followed where hydatid cysts were evacuated, and the superior capsule of the cyst was kept in place to separate the thoracic and abdominal compartments	Uneventful
Kahraman [8]	1	2017	40 years Male	Dyspnea and abdominal pain	Liver	Thoracotomy	Primary repair	Uneventful
Gunal *et al*. [9]	1	2016	68 years Female	Left-sided chest pain, fever and fatigue	Spleen	Thoracoabdominal	Primary repair	Uneventful
Oz *et al*. [10]	2	2014	35 years Male	Cough	Liver	Thoracotomy	Primary repair	Uneventful
			60 years Female	Dyspnea and abdominal swelling	Spleen	Thoracotomy	Primary repair	Uneventful
Chautems *et al*. [11]	5	2005	Median age of all included cases (35 pts) was 40 years, with a total of 20 females and 15 males	Abdominal pain was the most commonly reported symptom among all included pts	Liver	Laparotomy	Primary repair	13 of the total pts developed postoperative complications including brachial plexus injury, post-transfusion hepatitis C and other complications requiring medical treatment or drainage
Kilani *et a*l. [12]	23	2000	Among all included (37) pts, the mean age was 45.2 years, 22 were women and 15 were men	Lower chest pain and productive cough were the most frequent symptoms	Liver	Thoracotomy	Primary repair	11 of the 37 pts had complications including acute renal failure, sepsis, persistent biliary fistula, cholangitis, empyema, biliary fistula and death

*RUQ* right upper quadrant, *Pts* patients

The rarity of adult Bochdalek hernia was demonstrated in a retrospective study by Mullins *et al*., which showed an incidence of 0.17% [13]. The majority of cases occur on the left hemidiaphragm. Early embryogenic closure of the right pleuroperitoneal canal and the hepatic protection provided may explain this left-sided preponderance. Blunt or penetrating thoracoabdominal trauma is considered the most common causative factor, followed by iatrogenic injuries. Nontraumatic DH, on the other hand, might be attributed to persistent lung infections or factors related to the prolonged or sudden increase in intra-abdominal pressure. The absence of all known predisposing factors in the present case additionally supported a causative role for retroperitoneal hydatidosis.

It is not uncommon for ADH to remain clinically silent for years. In contrast to congenital DH, adult-onset herniations usually manifest as gastrointestinal symptoms rather than pulmonary symptoms. Abdominal pain was reported in 62% of cases and therefore considered the most frequent complaint [14]. Other possible symptoms include dyspnea, chest pain and vomiting. Due to the nonspecific symptomatology of ADH and its rarity, diagnosis may be delayed, resulting in serious complications such as incarceration and strangulation.

With respect to diagnostic modality, chest X-ray may demonstrate the presence of visceral herniation with the air-fluid appearance and/or contralateral deviation of the mediastinum; thus it is the initial test to perform and could be diagnostic in many cases such as ours. However, CT scan is the modality of choice to detect and visualize the hernia defect and its contents [15].

In the past, an operative approach was typically employed based on the chronicity of the condition, as it was believed that the release of adhesions of hernial content in the thorax would be difficult via transabdominal incision and safer via a transthoracic approach. Nevertheless, recently published experience in using the abdominal approach has shown its feasibility and safety, as demonstrated in a retrospective study by Payne *et al*. that reported a higher mortality rate with the thoracic than the abdominal approach [16]. Additionally, patients who underwent surgery using an abdominal approach were found to have a lower incidence of pneumonia than patients undergoing the classic transthoracic approach [17].

In fact, most emergency cases that require surgical intervention are done through an abdominal incision [13]. Kishore *et al*. reported a successful repair of DH through laparotomy in 88% of their series [18].

With the advancements in minimally invasive techniques, laparoscopic repair has been tried and proven to be a legitimate option for DH repair. Although shorter hospital stay and faster recovery are reported advantages of the laparoscopic technique, concerns regarding visceral injuries and recurrence rates were initially present. Liao *et al*. reviewed a total of 36 cases of DH that were repaired laparoscopically, with no reported recurrence (average follow-up was 15.1 months) [19]. Technical difficulties in the laparoscopic approach requiring conversion to open surgery have been reported in the literature, as in our case, where there was no posterior leaflet of the diaphragm in which to anchor the sutures [20].

There are not enough data on which to base a recommendation regarding methods of repair; while most reported cases used the primary suturing technique, 40% used mesh alone or in combination with suture repair [19]. Primary repair is seen to be the method of choice in traumatic/acute settings, as reported by Kishore *et al*., with only 11% requiring mesh placement [18].

## Conclusion

Hydatid disease continues to be a significant public health issue in several countries, including Saudi Arabia. The cysts’ ability to involve any site of the body makes diagnosis and management of resultant complications a challenge in some cases. ADH as a complication of hydatid disease remains a rare clinical entity. Retroperitoneal hydatidosis, in particular, is an exceptionally rare cause behind a similar presentation. To the best of authors' knowledge, this is the first reported case of ADH likely caused by retroperitoneal hydatid cysts. Knowledge about such existing causation would enable early detection and management to avoid serious complications. A multidisciplinary, individualized approach is recommended when managing similar cases to facilitate the provision of comprehensive care.

## Data Availability

Not applicable.

## Abbreviations

DH: Diaphragmatic hernia
CT: Computed tomography
HD: Hydatid disease
ADH: Acquired diaphragmatic hernia
